# Dataset for large-scale, lateral-torsional buckling tests of continuous beams in a grillage system

**DOI:** 10.1016/j.dib.2022.108532

**Published:** 2022-08-10

**Authors:** Ahmed Rageh, C. Shawn Sun, Daniel G. Linzell, Jay A. Puckett

**Affiliations:** a SDR Engineering Consultant Inc., Tallahassee, FL, USA; b Department of Civil Engineering and Construction Management, California State University, Northridge, CA, USA; c Department of Civil and Environmental Engineering, University of Nebraska-Lincoln, Lincoln, NE, USA; d Durham School of Architectural Engineering and Construction, University of Nebraska-Lincoln, Omaha, NE, USA

**Keywords:** Full-scale, Bridge, Steel, Stringer, Floorbeam, Lateral-torsional buckling, Timber, Struts

## Abstract

A number of large-scale tests of a grillage system are summarized and reduced data are provided. The tests were completed in association with a Louisiana Transportation Research Center (LTRC) project for the Louisiana Department of Transportation and Development (LA DOTD) whose goal was to better quantify behavior and limit states of steel bridge floor systems to refine longitudinal member (stringer) load-rating calculations. A number of tests focused on the behavior of the stringers resting on transverse members (a floorbeam), a system akin to a grillage. The system was proportioned with the expectation that stringer lateral-torsional buckling, a key steel bridge beam design and load-rating limit state, would occur. The provided dataset includes specimen descriptions and output from 58 tests. Investigated parameters included: stringer unbraced lengths; bracing types (i.e., bolted steel diaphragms versus clamped timber struts); load locations; and support conditions. Sample tests are described and reduced results summarized and presented.

**Specification Table**

**Table:** 

Subject	Steel beams, bridge floor systems
Specific subject area	Lateral-torsional buckling
Type of data	Tables Figures
How data were acquired	Voltage based strain gages and transducers, LVDTs, load and pressure cells acquired using National Instruments and Bridge Diagnostics, Inc. acquisition systems
Data format	Raw Reduced Excel spreadsheets
Description for data collection	Bracing locations and types; applied loads; flexible or rigid interior support; bolted or unbolted beams to interior support
Data source location	• Institition: University of Nebraska-Lincoln, Scott Engineering Center Structures Laboratory • City/State: Lincoln, Nebraska • Country: United States
Data accessibility	Repository name: Mendeley Data Direct URL to data: https://data.mendeley.com/datasets/frbrpzzf7g/2[1]
Related research article	Sun, C. S., Linzell, D. G., Puckett, J. A., Akintunde, E., & Rageh, A. (2022). Experimental Study of Continuous-Beam Lateral Torsional–Buckling Resistance with a Noncomposite Concrete Deck. Journal of Structural Engineering, 148(4), 04022026. [2]

**Value of the Data**

• This data set was collected by testing a full-scale grillage assembly to examine the lateral-torsional buckling strength of continuous stringers. A review of the literature indicated that this type of data was previously unavailable to researchers and structural engineers. It will allow researchers studying steel structures to access approximately 50 tests that study beam (i.e., stringer) behavior by accounting for various bracing configurations, boundary conditions, and load cases. It will also help bridge engineers understand bracing effects provided by intermediate steel diaphragms and by timber ties having minimal axial stiffness, and could contribute to solving challenges associated with load rating existing bridges.
• This data will be beneficial to researchers investigating lateral-torsional buckling behavior of steel beams. It will also benefit bridge engineers who perform load ratings of continuous steel stringers. They can study the behavior of stringers of various configuration, including having rigid and flexible interior supports, various levels of bracing provided by the intermediate steel diaphragms and timber ties, and bolted and unbolted connections to the floorbeam. They can compare data from different tests and understand influence of various parameters on beam behavior. The tests collected important data needed to examine beam buckling behavior, including applied loads, and stringer deflections and strains. Accompanying descriptions of test setups, instrumentation, and data processing will serve as a valuable reference for other researchers who wish to perform similar tests.

## 1 Data Description

The dataset contains a total of five subsets. Each subset contains test results of one test setup, and each test setup contains multiple test runs. Please refer to the next section for more details about test setups and test runs.

The provided data for each test run contains the test results arranged in Excel tabulated format and representative MATLAB figures. Each Excel table contains 85 columns, with each column representing one measured response time history. Descriptions of tabulated data columns are provided in Table 1.

**Table 1:** Tabulated test data description.

Table Column (s)	Description
A	Time in seconds
B:C	Applied loads at locations 3 and 10 in kips, respectively
D:S	Measured strains in microstrain at locations 3, 6, 7, and 10 at Top North (TN), Top South (TS), Bottom North (BN), and Bottom South (BS).
T:W	Measured lateral and vertical displacements in inches at locations 3 and 10, respectively.
X:BY	Measured strains in microstrain at the rest of instrumented locations with each location contains measurements from three strain sensors
BZ:CC	Measured strains in microstrain at the instrumented diaphragm locations
CD:CG	Load cells measurements in kips

The content of each sub dataset is described in Table 2 as a function of the test run number (i) with XXX denoting test descriptions as detailed in the following section.

**Table 2:** Sub-dataset content.

		Files	
Sub-dataset Folder	Test Run (i)	Name	Description
**Setup-1**	*i* = 1 to 8	XXX_TRi_Recordings.xls	Test run (i) excel data table with all test results
		XXX_TRi_RecordingsToPmax.xls	Test run (i) excel data table with test results up to the maximum applied load
		XXX_TRi_LoadDisplacement.fig	Test run (i) representative load-displacement MATLAB figure
		XXX_TRi_LoadStress.fig	Test run (i) representative load-stress MATLAB figure

**Setup-1’**	*i* = 1, 3, 5 and 7	XXX_TRi_Recordings.xls	Test run (i) excel data table with all test results
		XXX_TRi_RecordingsToPmax.xls	Test run (i) excel data table with test results up to the maximum applied load
		XXX_TRi_LoadDisplacement.fig	Test run (i) representative load-displacement MATLAB figure
		XXX_TRi_LoadStress.fig	Test run (i) representative load-stress MATLAB figure

**Setup-2**	*i* = 9 to 28	XXX_TRi_Recordings.xls	Test run (i) excel data table with all test results
		XXX_TRi_RecordingsToPmax.xls	Test run (i) excel data table with test results up to the maximum applied load
		XXX_TRi_LoadDisplacement.fig	Test run (i) representative load-displacement MATLAB figure
		XXX_TRi_LoadStress.fig	Test run (i) representative load-stress MATLAB figure

**Setup-2’**	*i* = 9, 11, 13, 15, 17, 19, 21, 23, 25, 27	XXX_TRi_Recordings.xls	Test run (i) excel data table with all test results
		XXX_TRi_RecordingsToPmax.xls	Test run (i) excel data table with test results up to the maximum applied load
		XXX_TRi_LoadDisplacement.fig	Test run (i) representative load-displacement MATLAB figure
		XXX_TRi_LoadStress.fig	Test run (i) representative load-stress MATLAB figure
**Setup-3**	*i* = 29 to 44	XXX_TRi_Recordings.xls	Test run (i) excel data table with all test results
		XXX_TRi_RecordingsToPmax.xls	Test run (i) excel data table with test results up to the maximum applied load
		XXX_TRi_LoadDisplacement.fig	Test run (i) representative load-displacement MATLAB figure
		XXX_TRi_LoadStress.fig	Test run (i) representative load-stress MATLAB figure

## 2 Experimental Design, Materials and Methods

A grillage assembly was fabricated, constructed, and tested in the University of Nebraska-Lincoln Scott Engineering Center Structures Laboratory to simulate bridge behavior. The assembly was designed so that the middle stringer would serve as the test specimen and be subjected to loads and boundary conditions closely matching those experienced in actual bridges. The test assembly and specimens are first described followed by instrumentation used to measure behavior.

### 2.1 Assembly and Specimens

A large-scale representation of a two-span grillage was tested to mimic a bridge floor system. The grillage contained three stringers supported on a floorbeam at midspan and by abutments at each end. All stringers were W16 × 31 (W410 × 46) rolled I-beams spaced laterally at 4.0 ft. (1.22 m) on-center. Each span was 24.0 ft. (7.3 m) center to center. Abutments at the end of the bridge that supported each stringer were W16 × 31 s (W410 × 46 s) and supported on the laboratory strong floor. The interior, transverse, floorbeam was a W24 × 68 (W610 × 101) cross section with a total length of 25.0 ft. (7.62 m). The floorbeam was supported by up to five supports, two at its ends and three underneath each stringer. These options enabled examination of the influence of either a “flexible” or a “stiff,” intermediate, transverse, supporting member on behavior.

**Fig. 1:**
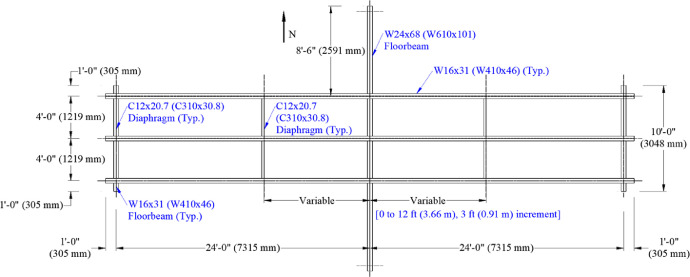
Framing plan.

**Fig. 2:**
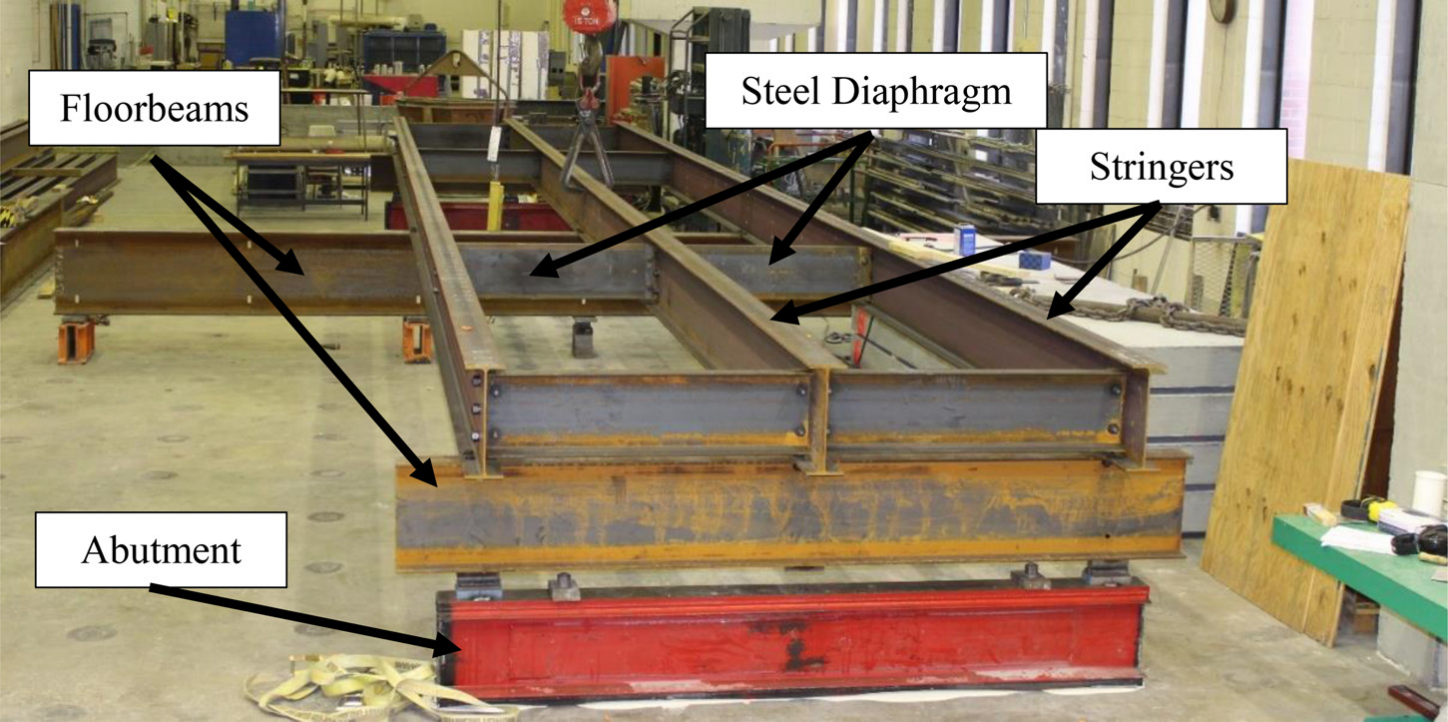
Assembled grillage.

Lateral support to the stringers was provided by transverse C12 × 20.7 (C310 × 30.8) diaphragms at the abutments, over the floorbeam, and at various unbraced lengths in each span. In addition, 4.0 in. x 4.0 in. (102 mm × 102 mm) timber struts were clamped to stringer top flanges at various unbraced lengths to investigate the influence of minimal lateral restraint on lateral-torsional buckling capacity. The framing plan is shown in Fig. 1, and the actual grillage assembly is shown in Fig. 2.

**Fig. 3:**
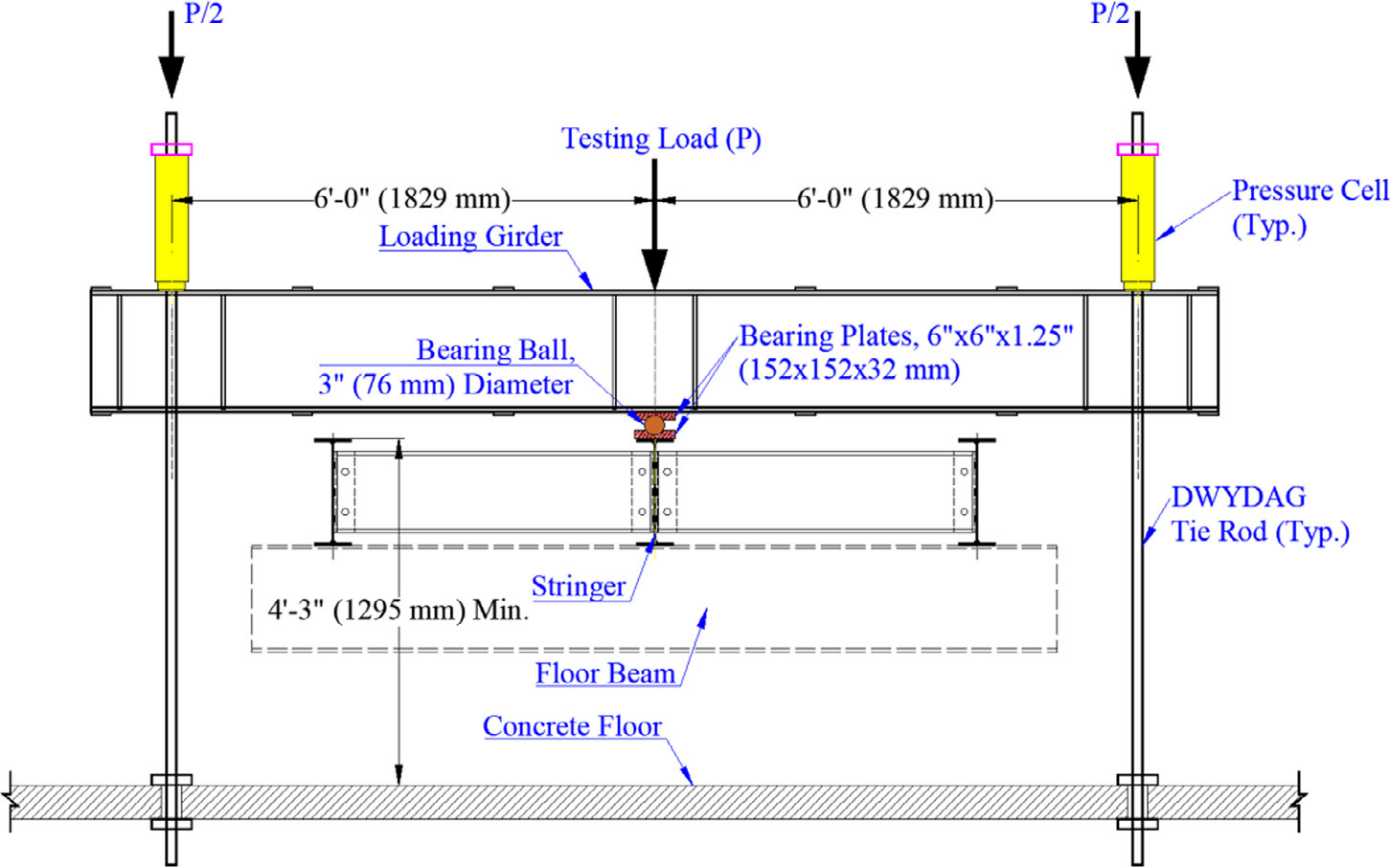
Loading system.

**Fig. 4:**
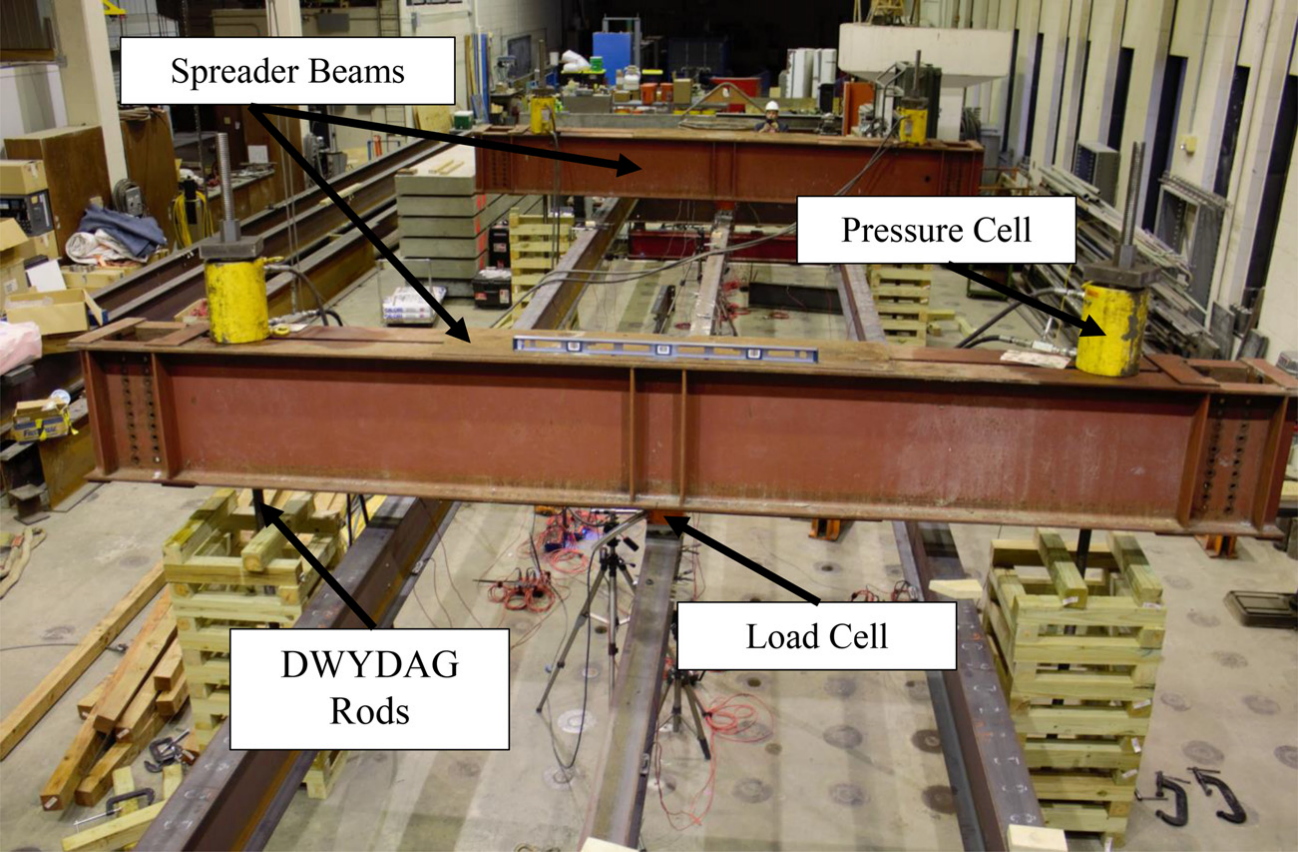
Final grillage assembly.

Point loads were applied at the midspan of one or both spans to induce different moment gradients in the middle stringer. Each point load was applied using hydraulic through-hole RAMs placed at the ends of the spreader beams. The RAMs applied equal tensile loads to 2.0-in. (51-mm) diameter DWYDAG rods connected to the strong floor, which applied downward force to the spreader beams and, ultimately, to the interior stringer. Loads were applied to the stringer's top flange using spherical bearings that mitigated resistance to buckling. An elevation view of the loading system is shown in Fig. 3, and the final laboratory assembly in Fig. 4.

### 2.2 Data Acquisition

Devices used for laboratory testing included data acquisition systems, Linear Variable Differential Transformers (LVDTs), load and pressure cells, strain gages, and strain transducers. One of the data acquisition systems was a National Instruments PXIe 1085, which had 16 hybrid slots, 1 PXI express system timing slot, and a 24 GB/s PXI chassis. Foil strain gages at critical locations on the interior stringer, and pressure and load cells were acquired by this system. Bridge Diagnostics Incorporated (BDI) ST350 strain transducers were used at other interior stringer locations. To measure stringer lateral and vertical displacements, BDI LVDTs were used. Strain transducers and LVDTs were connected to a BDI STS4-4 Wireless Intelliducer Node that transferred measured responses to a BDI STS4 wireless base station. The base station was accessed using a laptop and BDI's STS-LIVE application to store, reduce, and visualize measured response.

### 2.3 Instrumentation Plan

**Fig. 5:**
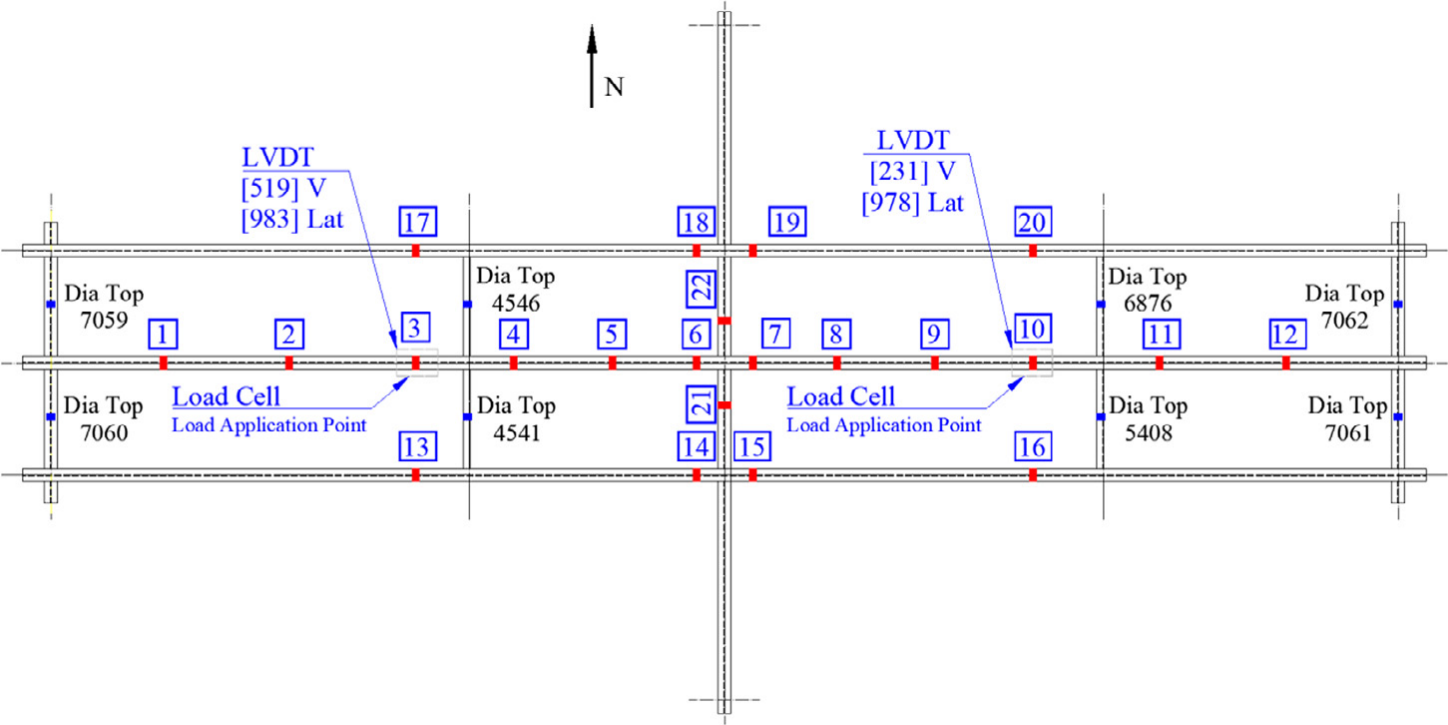
Plan view denoting instrumented section locations and sensor identifiers.

A combination of pressure and loads cells, strain gages, strain transducers, and LVDTs measured applied loads and grillage response. The interior stringer had 12 instrumented sections with 46 sensors, while exterior stringers were instrumented at four sections, each with 12 sensors. The floorbeam was instrumented in two sections using six sensors and the steel diaphragms at two sections with two sensors per section. An instrumentation plan is shown in Fig. 5.

**Fig. 6:**
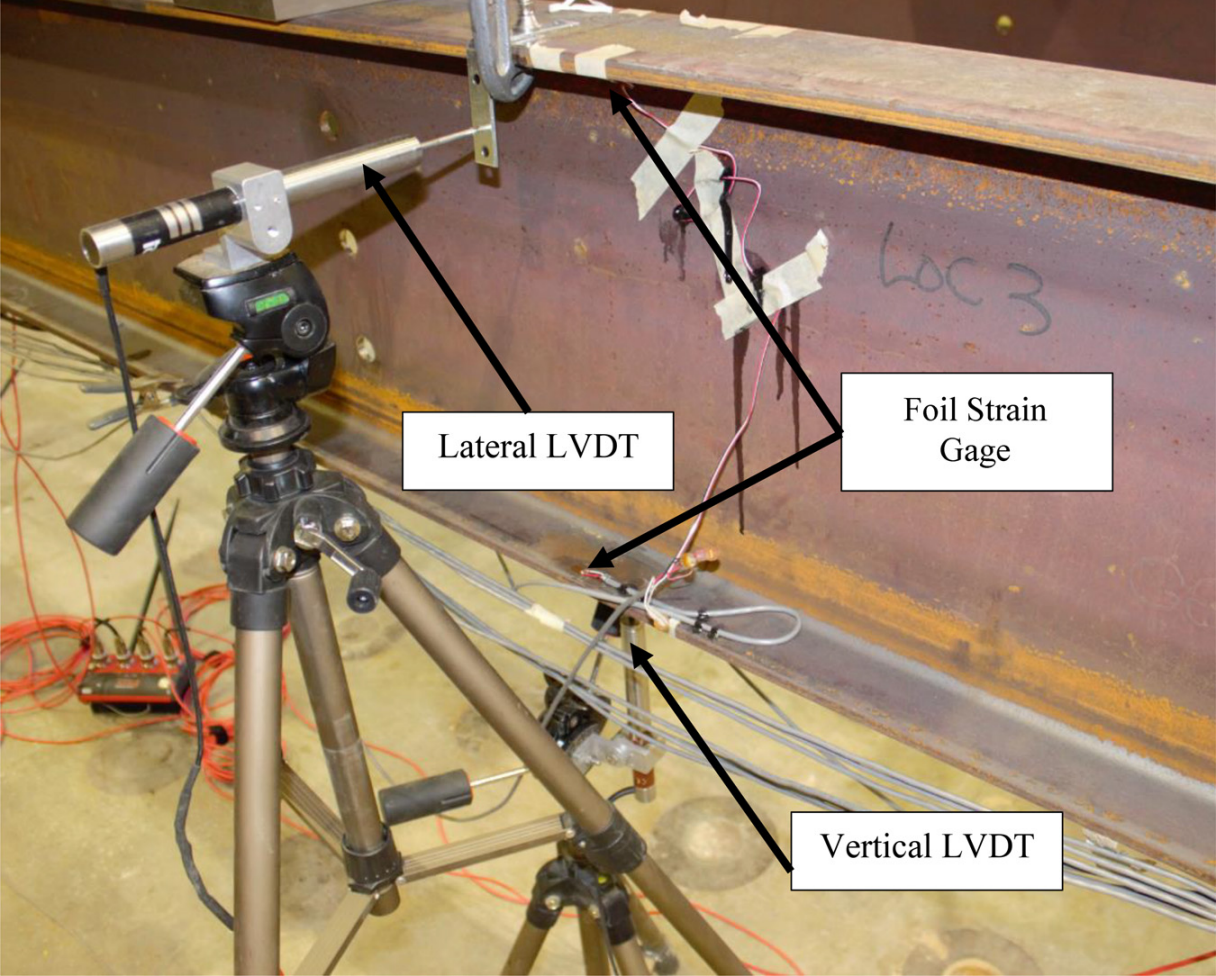
Section 3 instrumentation.

Interior stringer Sections 3, 6, 7, and 10 were instrumented with four foil strain gages at the top and bottom flange tips to measure major and minor bending. Point loads, measured using pressure and load cells, were applied at Sections 3 and 10. Vertical and lateral displacements were measured at interior stringer Sections 3 and 10 using LVDTs. Section 3 foil gage and LVDT locations are shown in Fig. 6(a-b).

**Fig. 7:**
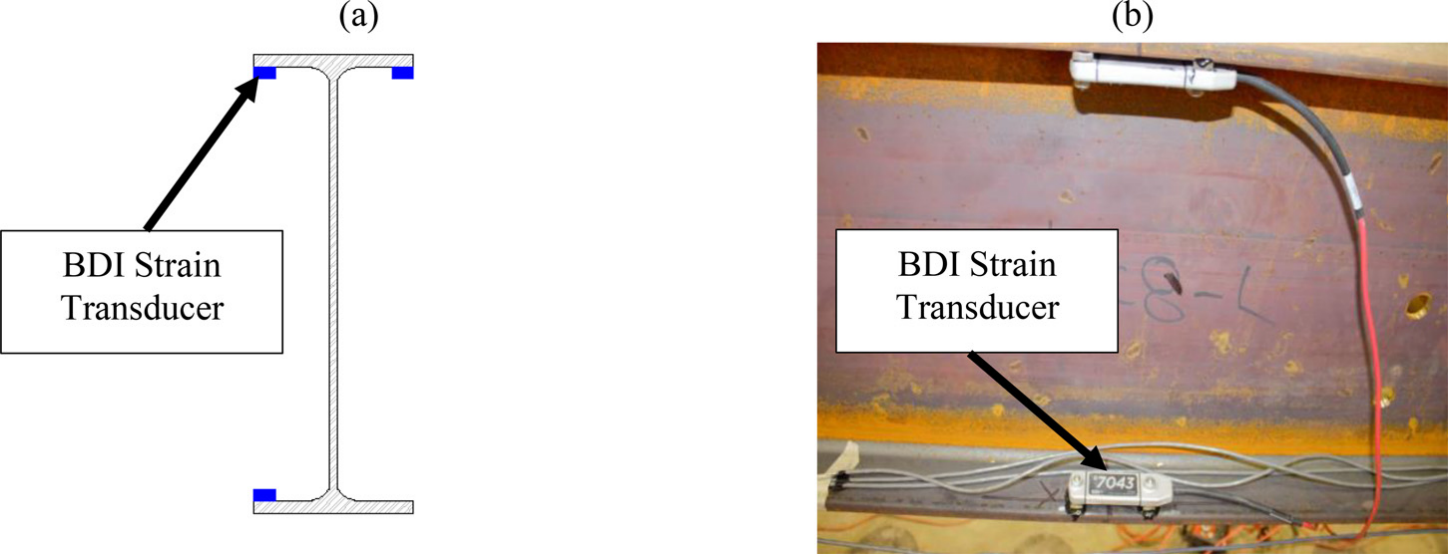
Section 8 instrumentation: (a) sketch; (b) photo.

**Fig. 8:**
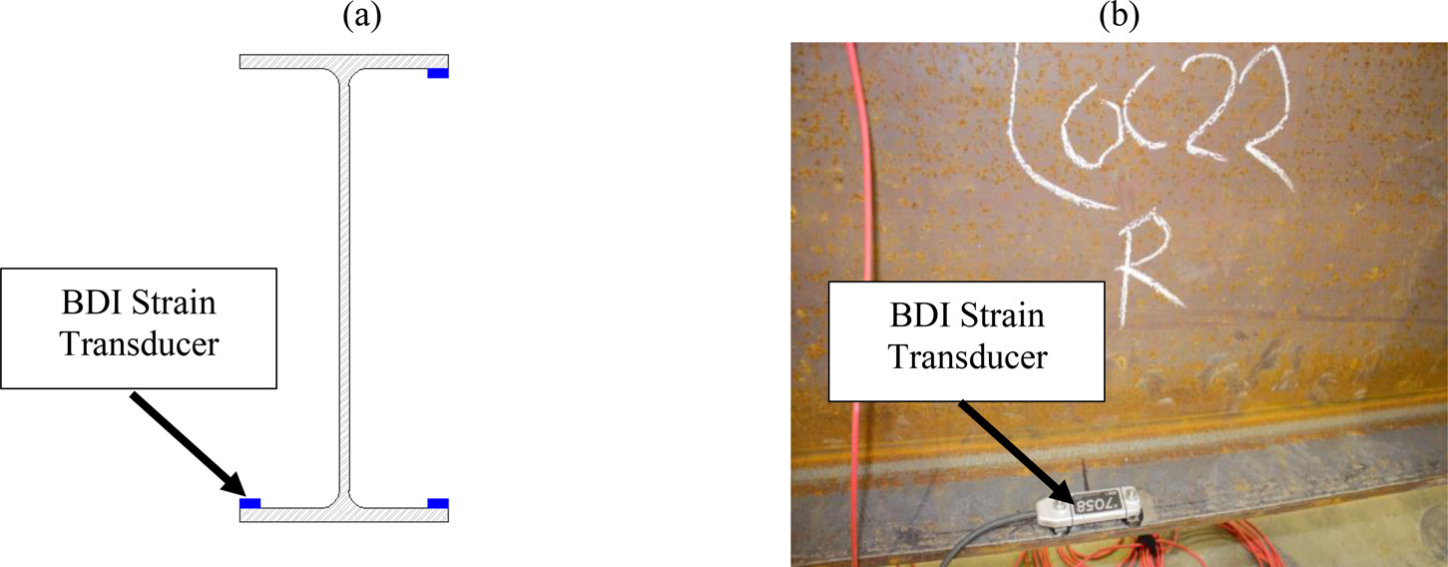
Section 22 instrumentation: (a) sketch; (b) photo.

**Fig. 9:**
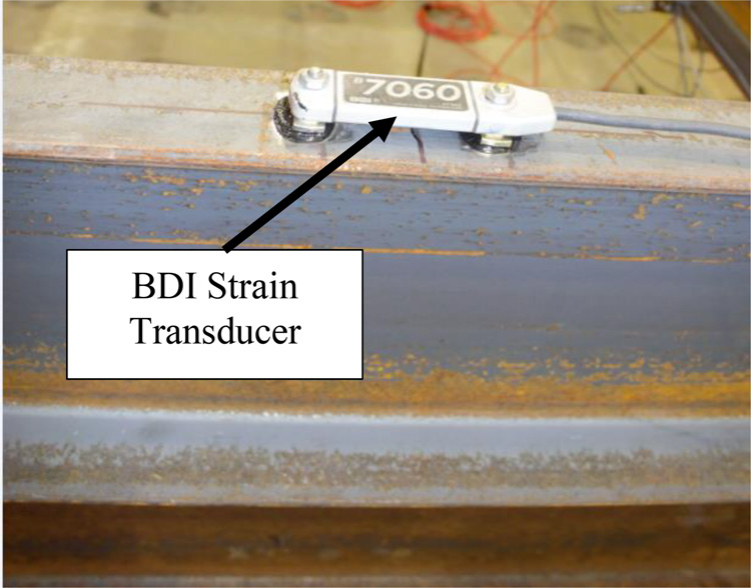
Steel diaphragm instrumentation.

The remaining stringer and interior floorbeam sections were instrumented using three Bridge Diagnostics, Inc. strain sensors. Fig. 7(a-b) indicates that stringers were instrumented at each section with two sensors at the tension flange tips and one sensor at the compression flange tip, with tension and compression zones switching between the top and bottom flanges as a function of the expected bending moment diagram. Sections 5 to 8, 14 to 15, and 18 to 19 were assumed to experience negative bending. Fig. 8(a-b) indicates that each floorbeam section was instrumented with two sensors at the bottom flange tips and one at the top flange tip. Steel diaphragms were instrumented at midspan with BDI strain sensors at the mid-width of the top flange (Fig. 9).

### 2.4 Test Categories and Configurations

**Fig. 10:**
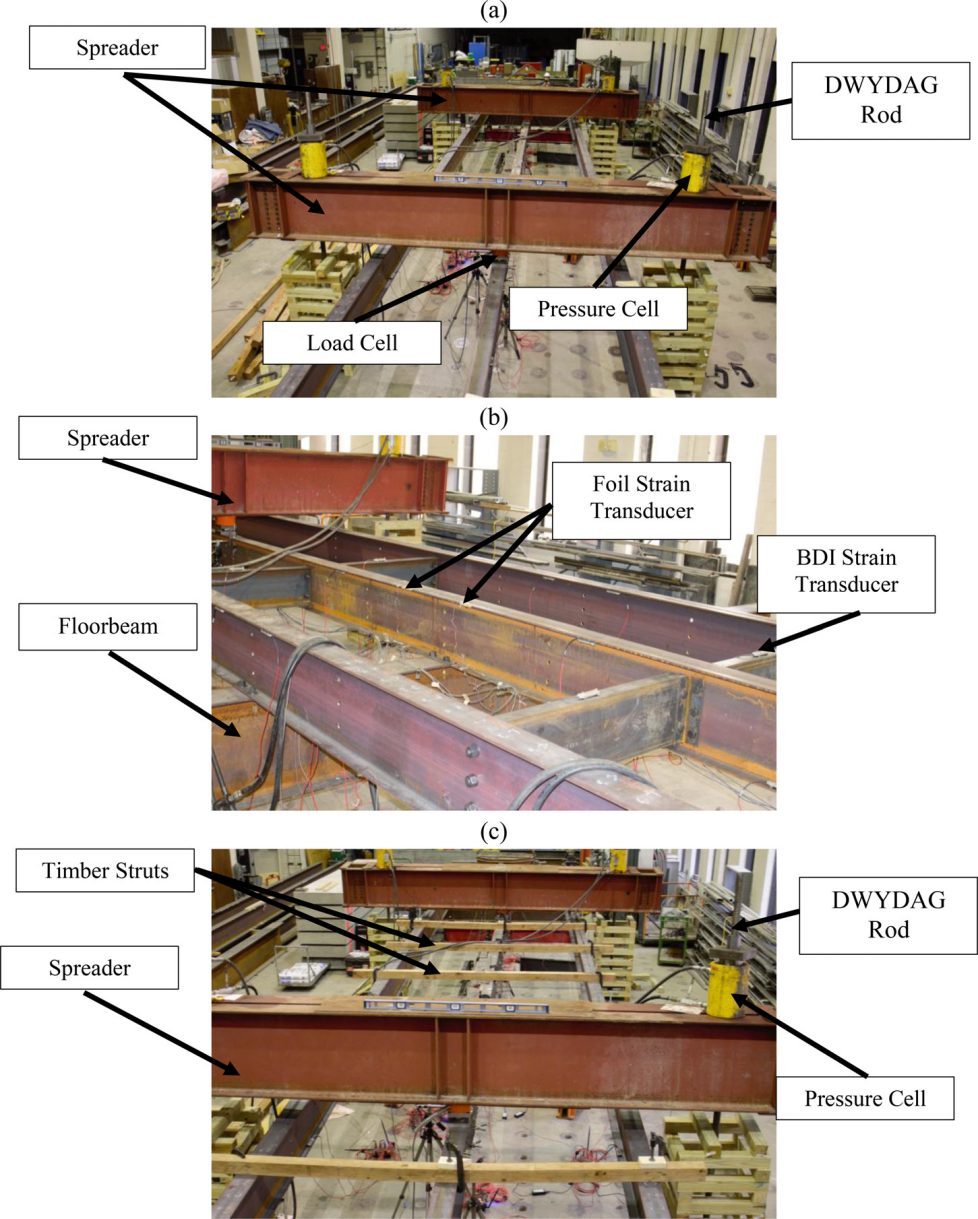
Testing categories: (a) Setup-1; (b) Setup-2; (c) Setup-3.

Tests fell into three general categories: (i) no interior diaphragms (Setup-1), Fig. 10(a); (ii) braced using interior diaphragms (Setup-2), Fig. 10(b); and (iii) top flange braced using timber struts (Setup-3), Fig. 10(c). Each category involved multiple test runs encompassing the following parameters:

1. Rigid interior floorbeam (R) (i.e., supported at five locations along the length) or flexible interior floorbeam (F) (i.e., supported at ends), see Fig. 11(a-b);

**Fig. 11:**
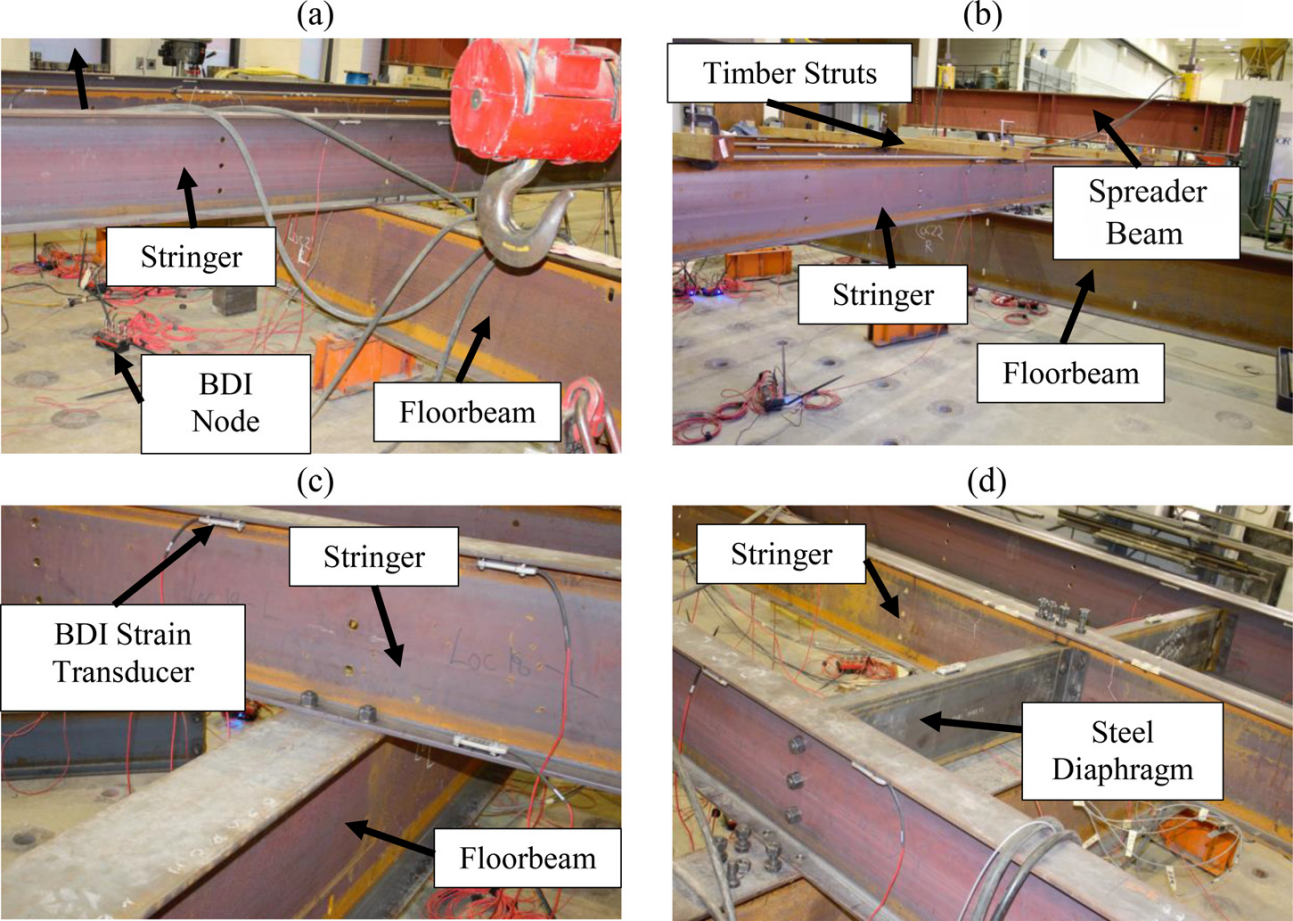
Testing parameters: (a) R; (b) F; (c) B; (d) NB.
2. Bolted stringer to interior floorbeam top flange (B) or unbolted stringer to interior floorbeam (NB), see Fig. 11(c-d);
3. Single point load applied at Section 3 (1PL) or two-point loads at Sections 3 and 10 (2PL);
4. Varying steel diaphragm locations (i.e., full depth unbraced lengths); and
5. Varying timber strut locations (i.e., top flange unbraced lengths).

**Fig. 12:**
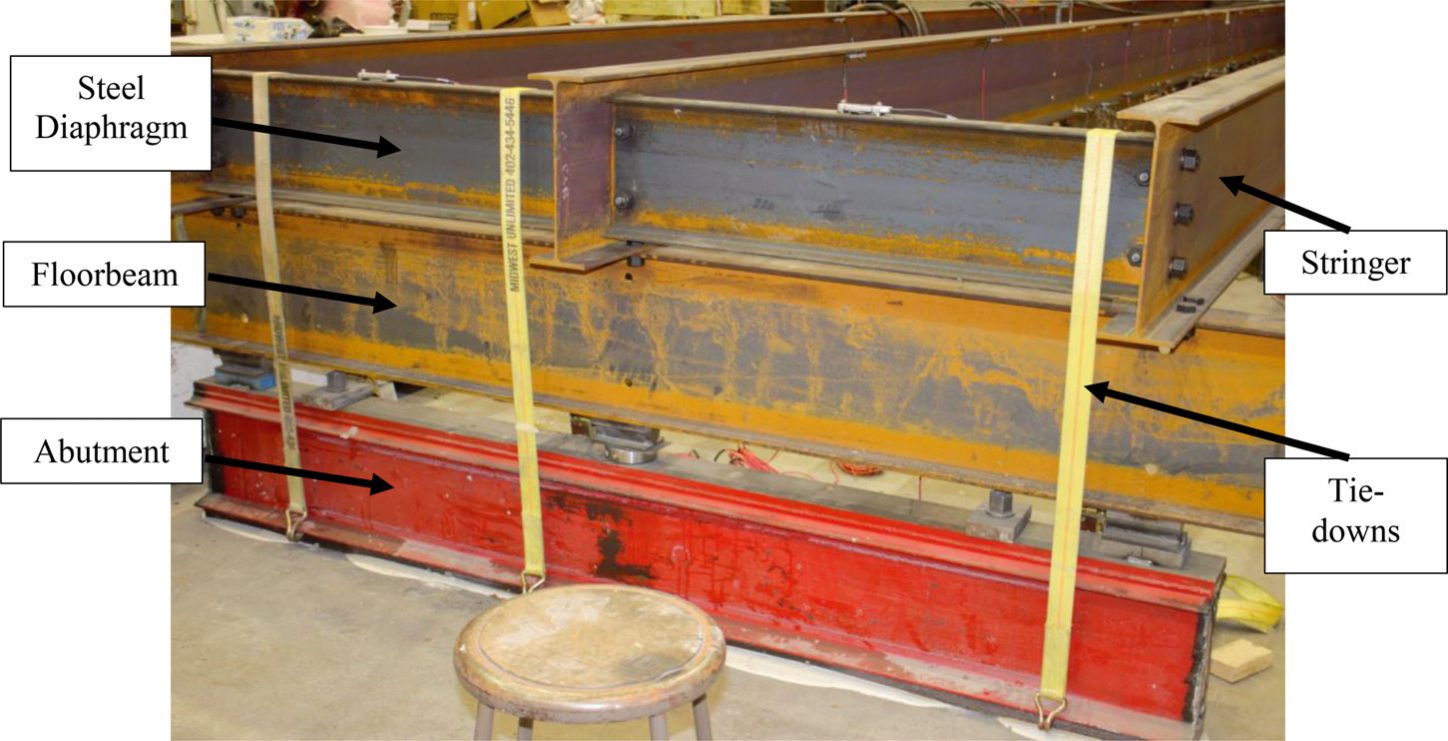
Tie-downs.

Setup-1 and Setup-2 involved testing the system under 1PL and 2PL. To investigate the influence of stringer continuity on lateral-torsional buckling, Setup-1 and Setup-2 1PL tests were performed twice. The first set of tests did not utilize tie-downs at the abutment supporting the unloaded span and, as a result, uplift occurred and stringers acted as though they were simply-supported in the loaded span. The second set of test runs included tie-downs at the abutment, and the stringers behaved as 2-span, continuous beams (Fig. 12). Test runs results with tied down east ends are referred to herein as Setup-1’ and Setup-2’.

### 2.5 Test Matrix

An annotated Microsoft Excel file is provided for each of the 58 tests at the online source associated with this manuscript. The tables provided below contain brief descriptions of individual tests. Table 3 lists the description of each symbol used in the ID of each test run listed in Tables 4 to 6. Setup-1 and Setup-1’ tests are provided in Table 4, Setup-2 and Setup-2’ in Table 5, and Setup-3 in Table 6.

**Table 3:** Test ID symbol descriptions.

Symbol	Description
R	Rigid floorbeams (i.e., 5 supports along the length)
F	Flexible floorbeam (i.e., 2 supports at ends)
B	Braced stringer bottom flange (i.e., stringer bottom flange bolted to interior floorbeam top flange)
NB	Not Braced stringer bottom flange (i.e., stringer bottom flange not bolted to floorbeam top flange)
SD	Stringer braced with Single steel Diaphragm over interior floorbeam
18D, 14D, 38D or 12D	Stringers braced at 2 locations with steel Diaphragms at a spacing of 1/8, 1/4, 3/8, or 1/2 of the span length as measured from the interior floorbeam
12TS, 13TS, 14TS or 15TS	Stringer top flanges braced at multiple locations with Timber Struts spaced at 1/2, 1/3, 1/4, or 1/5 of the span length
1PL	Interior stringer loaded with 1 Point Load in a span
2PL	Interior stringer loaded with 2 Point Loads; each span with 1 point load.

**Table 4:** Setup-1, 1’.

Test	Setup	ID	Bracing condition
**1**	**Setup-1**	R-NB-1PL	None
**2**		R-NB-2PL	
**3**		R-B-1PL	
**4**		R-B-2PL	
**5**		F-NB-1PL	
**6**		F-NB-2PL	
**7**		F-B-1PL	
**8**		F-B-2PL	

**1’**	**Setup-1’**	R-NB-1PL	None
**3’**		R-B-1PL	
**5’**		F-NB-1PL	
**7`**		F-B-1PL

**Table 5:** Setup-2, 2’.

Test	Setup	ID	Bracing condition
**9**	**Setup-2**	R-NB-SD-1PL	1 diaphragm over interior floorbeam
**10**		R-NB-SD-2PL	1 diaphragm over interior floorbeam
**11**		R-NB-12D-1PL	2 steel diaphragms at span/2 from floorbeam
**12**		R-NB-12D-2PL	2 steel diaphragms at span/2 from floorbeam
**13**		R-NB-18D-1PL	2 steel diaphragms at span/8 from floorbeam
**14**		R-NB-18D-2PL	2 steel diaphragms at span/8 from floorbeam
**15**		R-NB-14D-1PL	2 steel diaphragms at span/4 from floorbeam
**16**		R-NB-14D-2PL	2 steel diaphragms at span/4 from floorbeam
**17**		R-NB-38D-1PL	2 steel diaphragms at 3 span/8 from floorbeam
**18**		R-NB-38D-2PL	2 steel diaphragms at 3 span/8 from floorbeam
			
**19**		R-B-SD-1PL	1 steel diaphragm over interior floorbeam
**20**		R-B-SD-2PL	1 steel diaphragm over interior floorbeam
**21**		R-B-12D-1PL	2 steel diaphragms at span/2 from floorbeam
**22**		R-B-12D-2PL	2 steel diaphragms at span/2 from floorbeam
**23**		R-B-18D-1PL	2 steel diaphragms at span/8 from floorbeam
**24**		R-B-18D-2PL	2 steel diaphragms at span/8 from floorbeam
**25**		R-B-14D-1PL	2 steel diaphragms at span/4 from floorbeam
**26**		R-B-14D-2PL	2 steel diaphragms at span/4 from floorbeam
**27**		R-B-38D-1PL	2 steel diaphragms at 3 span/8 from floorbeam
**28**		R-B-38D-2PL	2 steel diaphragms at 3 span/8 from floorbeam

**9’**	**Setup-2’**	R-NB-SD-1PL	1 steel diaphragm over interior floorbeam
**11’**		R-NB-12D-1PL	2 steel diaphragms at span/2 from floorbeam
**13’**		R-NB-18D-1PL	2 steel diaphragms at span/8 from floorbeam
**15’**		R-NB-14D-1PL	2 steel diaphragms at span/4 from floorbeam
**17’**		R-NB-38D-1PL	2 steel diaphragms at 3 span/8 from floorbeam
			
**19’**		R-B-SD-1PL	1 steel diaphragm over interior floorbeam
**21’**		R-B-12D-1PL	2 steel diaphragms at span/2 from floorbeam
**23’**		R-B-18D-1PL	2 steel diaphragms at span/8 from floorbeam
**25’**		R-B-14D-1PL	2 steel diaphragms at span/4 from floorbeam
**27’**		R-B-38D-1PL	2 steel diaphragms at 3 span/8 from floorbeam

**Table 6:** Setup-3.

Test	Setup	ID	Bracing condition
**29**	**Setup-3**	R-NB-12TS-2PL	Timber struts at span/2
**30**		R-NB-13TS-2PL	Timber struts at span/3
**31**		R-NB-14TS-2PL	Timber struts at span/4
**32**		R-NB-15TS-2PL	Timber struts at span/5
			
**33**		R-B-12TS-2PL	Timber struts at span/2
**34**		R-B-13TS-2PL	Timber struts at span/3
**35**		R-B-14TS-2PL	Timber struts at span/4
**36**		R-B-15TS-2PL	Timber struts at span/5
			
**37**		F-NB-12TS-2PL	Timber struts at span/2
**38**		F-NB-13TS-2PL	Timber struts at span/3
**39**		F-NB-14TS-2PL	Timber struts at span/4
**40**		F-NB-15TS-2PL	Timber struts at span/5
			
**41**		F-B-12TS-2PL	Timber struts at span/2
**42**		F-B-13TS-2PL	Timber struts at span/3
**43**		F-B-14TS-2PL	Timber struts at span/4
**44**		F-B-15TS-2PL	Timber struts at span/5

### 2.6 Sample Comparisons

**Fig. 13:**
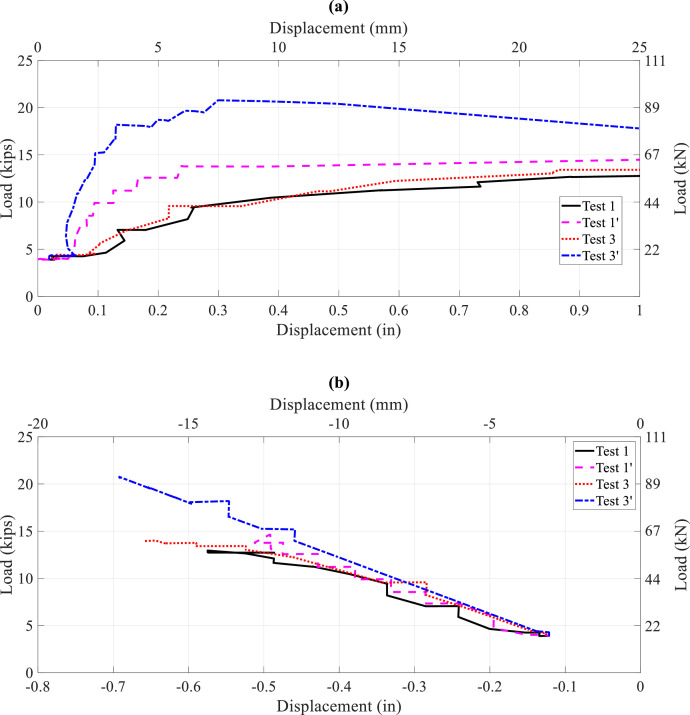
Load-displacement curves, single point load at Section 3, Tests 1, 1’, 3, and 3’: (a) lateral displacement at Section 3; (b) vertical displacement at Section 3

**Fig. 14:**
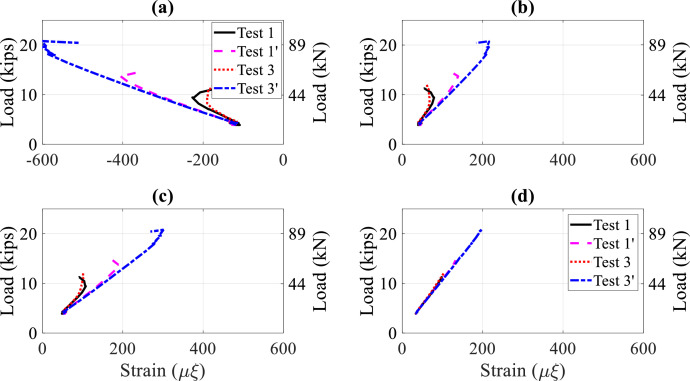
Load-strain curves, single point load at Section 3, Tests 1, 1’, 3 and 3’, top north strains at: (a) Section 3; (b) Section 6; (c) Section 7; (d) Section 10

Sample comparisons between three tests are presented to illustrate the influence of stringer continuity (i.e., tie-down effect), floorbeam flexibility (i.e., R or F), and stringer bottom flange bracing (i.e., B or NB) on stringer lateral-torsional buckling capacity. Applied load and lateral and vertical displacements are compared in Fig. 13 for test numbers 1 (R-NB-1PL), 1’ (R-B-1PL), 3 (R-NB-1PL), and 3’ (R-B-1PL) as described in Table 4. Tests 1’ and 3’ experienced higher buckling loads than Tests 1 and 3 due to the tie-down, which shows the influence of span continuity on lateral-torsional bucking capacity. Fig. 14 provides load versus longitudinal strain curves at 4 sections along the interior stringer for the tests. Tests 1’ and 3’ experienced larger strains before buckling.

**Fig. 15:**
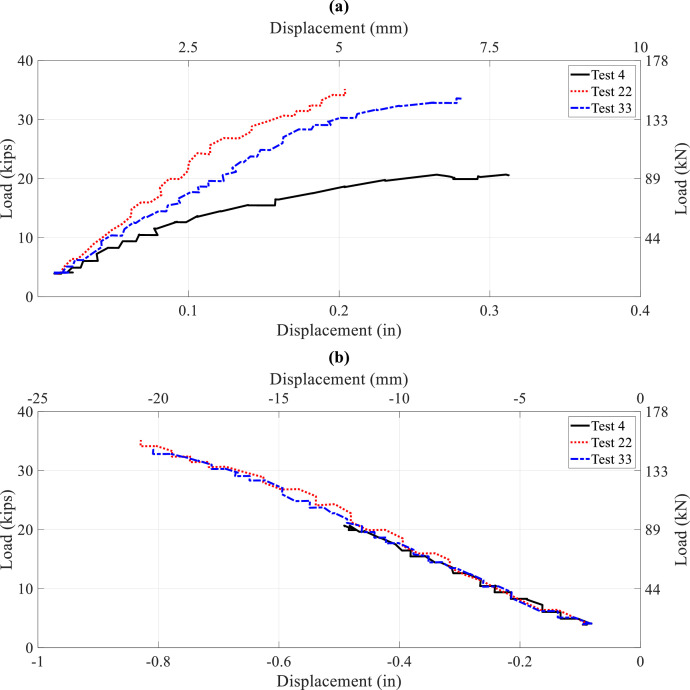
Load-displacement curves, single point load at Section 3, Tests 4, 22, and 33: (a) lateral displacement at Section 3; (b) vertical displacement at Section 3

**Fig. 16:**
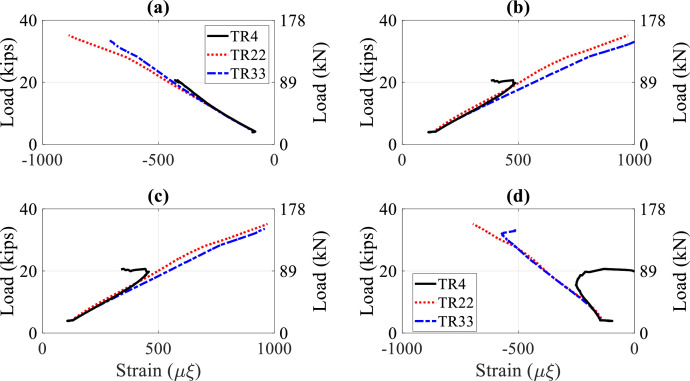
Load-strain curves, single point load at Section 3, Tests 4, 22 and 33, top north strains at: (a) Section 3; (b) Section 6; (c) Section 7; (d) Section 10

To illustrate bracing effects on lateral-torsional buckling capacity provided by interior steel diaphragms or timber struts, comparisons between Tests 4, 22, and 33 from Tables 4–6 are shown in Figs. 15 and 16. Load displacement comparisons in Fig. 15 indicate that braced stringers with a steel diaphragm attached to the web or, importantly, a timber strut clamped to the top flange increased buckling load significantly. Nondimensional ratios for the buckling loads, which compared values for Tests 22 and 33 to those for unbraced stringer Test 4, were 1.75 and 1.60. Load-strain curves at the top flange north side at Sections 3, 6, 7, and 10 as shown in Fig. 16 also reflected increased buckling capacity from steel diaphragms or timber struts at span/2 when compared to Test 4.

## Ethics Statement

The authors affirm they did not involve any human subjects or animal experiments associated with this research. The authors also affirm that they did not involve any data collected from social media platforms.

## CRediT authorship contribution statement

**Ahmed Rageh:** Conceptualization, Resources, Validation, Data curation, Writing – original draft. **C. Shawn Sun:** Conceptualization, Validation, Data curation, Writing – original draft. **Daniel G. Linzell:** Validation, Conceptualization, Supervision, Data curation, Writing – original draft. **Jay A. Puckett:** Validation, Conceptualization, Supervision, Data curation, Writing – original draft.

## Declaration of Competing Interest

The authors declare that they have no known competing financial interests or personal relationships that could have appeared to influence the work reported in this paper.

## Data Availability

Dataset for large-scale, lateral-torsional buckling tests of continuous beams (Original data) (Mendeley Data).
